# Diabetic Ketoacidosis in an Undiagnosed Type 1 Diabetic: A Case Study Highlighting Barriers to Rural Healthcare Access

**DOI:** 10.7759/cureus.79424

**Published:** 2025-02-21

**Authors:** Mohamman S Alhameed, Camila F Rocha

**Affiliations:** 1 Department of Biochemistry and Biophysics, University of Michigan, Ann Arbor, USA; 2 Department of Community and Rural Health, Great Plains Health Equity Institute, Des Moines, USA

**Keywords:** delayed diagnosis, diabetic ketoacidosis, first-generation students, healthcare disparities, health literacy, mentorship programs, rural healthcare, telemedicine access, type 1 diabetes, underserved communities

## Abstract

This case report describes a 17-year-old male from a rural, medically underserved community who presented to the emergency department with severe dehydration, altered mental status, and labored breathing. He was found to have new-onset type 1 diabetes mellitus complicated by diabetic ketoacidosis (DKA). Due to limited healthcare access, his symptoms were initially misattributed to a viral illness, delaying appropriate diagnosis and treatment. The case highlights the barriers faced by rural populations in obtaining timely medical care and underscores the importance of mentorship programs in increasing health literacy and provider outreach in these communities.

## Introduction

Healthcare disparities in rural communities remain a pressing issue, with limited access to specialists, long travel distances, and a shortage of primary care providers [1]. These barriers disproportionately affect populations with lower socioeconomic status, leading to worse health outcomes and delayed diagnoses [2]. Undiagnosed type 1 diabetes mellitus (T1DM) can lead to life-threatening complications such as diabetic ketoacidosis (DKA), particularly in populations with low health literacy and inadequate healthcare access [3]. Studies show that rural residents have higher rates of undiagnosed chronic illnesses due to fewer preventive care visits and limited access to endocrinologists [4,5].

Mentorship programs have emerged as a promising intervention to address healthcare gaps in underserved communities [6]. Health literacy, defined as the ability to obtain, process, and understand health information to make informed decisions, is often low in rural populations, contributing to worse outcomes [7]. First-generation medical students and healthcare professionals from similar backgrounds can serve as mentors, helping to improve health literacy and facilitate earlier engagement with the healthcare system [8]. Community outreach initiatives that focus on diabetes education, early screening, and telemedicine can help bridge existing disparities [9,10]. This case study illustrates the impact of these barriers on patient outcomes and discusses potential solutions through mentorship programs that bridge gaps in healthcare education and outreach.

## Case presentation

A 17-year-old male, the eldest son of farmworkers and a first-generation high school graduate, was brought to the emergency department by his mother with a three-week history of progressive fatigue, polydipsia, polyuria, and an unintentional 12-pound weight loss. His symptoms were initially dismissed by local urgent care providers as a viral infection, as the clinic lacked on-site laboratory testing and had limited experience with pediatric diabetes diagnosis. The family, residing in a healthcare shortage area, lacked reliable transportation, delaying further evaluation.

Upon presentation, the patient was tachypneic (respiratory rate: 32/min), tachycardic (heart rate: 118 bpm), and hypotensive (BP: 94/58 mmHg). A capillary blood glucose measurement was 480 mg/dL, and a venous blood gas revealed a pH of 7.12 with elevated anion gap metabolic acidosis, confirming DKA. He was admitted to the intensive care unit and managed with intravenous fluids, insulin therapy, and electrolyte replacement.

Further investigation revealed that the patient had never been screened for diabetes despite presenting with symptoms over the past several months. His mother, unaware of the warning signs, had been treating his fatigue with home remedies. This case underscores the role of health literacy in preventing severe complications and the need for accessible community education programs [11].

During his hospital stay, the patient required continuous insulin infusion for 48 hours before transitioning to subcutaneous insulin. Electrolyte imbalances were corrected over several days, and his metabolic status stabilized. A multidisciplinary team, including endocrinologists, nutritionists, and social workers, provided education on diabetes management. However, discussions revealed that the patient's family faced financial constraints that could limit access to insulin and glucose monitoring supplies after discharge.

To address this, the hospital's social services team connected the patient with a rural health program offering subsidized insulin and supplies. In addition, he was enrolled in a telemedicine-based mentorship program where first-generation medical students provided culturally competent diabetes education. This initiative helped improve adherence to insulin therapy and lifestyle modifications, mitigating the risk of recurrent DKA episodes.

## Discussion

The delay in diagnosing type 1 diabetes in rural communities is often multifactorial, involving structural, economic, and social barriers. Limited healthcare access in rural areas is a significant issue, as primary care clinics are scarce, and specialized care such as endocrinology is often unavailable [4,12]. A national survey found that rural patients with diabetes are 50% more likely to experience diagnostic delays compared to urban patients due to limited specialist availability [13]. This patient had no prior routine medical check-ups, a common issue in underserved regions where preventive care is deprioritized due to cost and accessibility concerns [5,14]. Many families in these areas rely on home remedies or delay seeking professional care until symptoms become severe, exacerbating preventable conditions [6,14]. In addition, rural hospitals and clinics often lack the necessary diagnostic tools, further delaying the proper identification and treatment of chronic conditions [10].

Health literacy plays a crucial role in diabetes management, but rural communities often lack the resources to educate individuals on recognizing early symptoms of chronic diseases [12,15]. Without proper understanding, families may not associate increased thirst, frequent urination, and weight loss with the onset of diabetes, leading to life-threatening complications like DKA [9]. A national study found that low health literacy is associated with a fourfold increase in preventable diabetes-related hospitalizations, underscoring the need for targeted education initiatives [16]. Many patients, especially in immigrant or low-income communities, may also be hesitant to seek medical care due to language barriers, mistrust in the healthcare system, or concerns over medical costs [10,16]. Culturally competent educational programs tailored to these populations can be instrumental in improving awareness and promoting early intervention.

Financial and transportation barriers further compound these challenges. Many uninsured or underinsured individuals cannot afford regular physician visits, let alone the long-term costs associated with chronic disease management [11,16]. The lack of reliable transportation also means that even when symptoms are recognized, patients may not be able to travel the long distances required to access specialized care [12]. Furthermore, in many cases, parents of young patients are forced to take unpaid leave or make significant financial sacrifices to seek medical attention for their children, further exacerbating their economic hardships [13]. Telemedicine and mobile clinics have shown promise in bridging this gap by providing virtual consultations and localized screenings, offering a sustainable model to improve healthcare access in remote areas [9,17].

To address these systemic issues, mentorship programs involving first-generation medical students and healthcare professionals can help foster trust and provide essential healthcare education to underserved communities [6,8]. By offering culturally relevant guidance and acting as patient advocates, mentors can bridge the gap between rural populations and medical providers, ultimately improving patient outcomes. Additionally, establishing partnerships between medical institutions and rural community centers can facilitate early screenings and intervention programs, helping to mitigate the progression of preventable diseases [7]. Expanding these initiatives and integrating them with broader healthcare infrastructure reforms, such as increasing funding for rural clinics and promoting policies that support affordable healthcare access, is essential to reducing disparities and preventing avoidable medical emergencies like DKA.

This case underscores the urgent need for systemic change in rural healthcare accessibility. While immediate interventions such as mentorship programs, telemedicine, and mobile clinics are effective in addressing acute barriers, long-term solutions require policy-level efforts to expand healthcare coverage, subsidize medical expenses for low-income populations, and improve the distribution of healthcare professionals in rural areas. A comprehensive, multi-faceted approach is necessary to ensure that patients in medically underserved communities receive the timely and effective care they need to manage chronic conditions like type 1 diabetes.

## Conclusions

Healthcare disparities in rural communities pose significant risks, particularly for preventable conditions like type 1 diabetes. The delayed diagnosis and subsequent development of DKA in this patient highlight the urgent need for improved access to healthcare services, early screening programs, and health literacy initiatives. Identifying and addressing barriers such as limited healthcare facilities, financial constraints, and low awareness is crucial in preventing similar life-threatening complications. Expanding mentorship programs, telemedicine, and community-based interventions can play a pivotal role in bridging these gaps and ensuring timely medical intervention. This case study underscores the importance of ongoing efforts to improve rural healthcare infrastructure and emphasizes the need for further research into effective strategies for mitigating healthcare disparities in underserved populations.
